# Modelling the micro- and macro- environment of pancreatic cancer: from patients to pre-clinical models and back

**DOI:** 10.1242/dmm.050624

**Published:** 2024-04-19

**Authors:** Eloise G. Lloyd, Joaquín Araos Henríquez, Giulia Biffi

**Affiliations:** University of Cambridge, Cancer Research UK Cambridge Institute, Robinson Way, Cambridge CB2 0RE, UK

**Keywords:** Macro-environment, Micro-environment, Pancreatic ductal adenocarcinoma, Preclinical *in vivo* models

## Abstract

Pancreatic ductal adenocarcinoma (PDAC) is a lethal malignancy with very low survival rates. Over the past 50 years, improvements in PDAC survival have significantly lagged behind the progress made in other cancers. PDAC’s dismal prognosis is due to typical late-stage diagnosis combined with lack of effective treatments and complex mechanisms of disease. We propose that improvements in survival are partly hindered by the current focus on largely modelling and targeting PDAC as one disease, despite it being heterogeneous. Implementing new disease-representative pre-clinical mouse models that capture this complexity could enable the development of transformative therapies. Specifically, these models should recapitulate human PDAC late-stage biology, heterogeneous genetics, extensive non-malignant stroma, and associated risk factors and comorbidities. In this Perspective, we focus on how pre-clinical mouse models could be improved to exemplify key features of PDAC micro- and macro- environments, which would drive clinically relevant patient stratification, tailored treatments and improved survival.

## Teaser

**Summary:** The micro- and macro- environments of pancreatic cancer are complex. Better recapitulation of this heterogeneity in pre-clinical mouse models is essential to improve treatment options for this dismal disease.

## Introduction

Pancreatic ductal adenocarcinoma (PDAC) is a complex and heterogenous disease with a dismal 5-year survival rate prognosis of ∼10-13% (Siegel et al., 2024). The presence of an extensive non-malignant stroma and distinct genetic profiles contribute to PDAC heterogeneity. Ageing, prolonged pancreas inflammation (i.e. chronic pancreatitis), obesity and type 2 diabetes increase the risk of developing the disease (Klein, 2021). However, diagnosis is hindered by the lack of screening options and absence of clear symptoms until the disease has progressed to an advanced stage. Consequently, few patients are eligible for potentially curative resection surgery, as most diagnoses occur when the disease is already metastatic. Other treatment options are limited to largely ineffective chemotherapy (Halbrook et al., 2023).

We believe that development of successful treatments relies on pre-clinical models that recapitulate not only human PDAC progression but also its heterogeneity and therapy response. In this Perspective, we discuss current challenges in modelling PDAC and ways to address them. While pre-clinical models for early-detection biomarker discovery and screening strategies for high-risk groups are also key, here we focus on pre-clinical mouse models (Box 1) that could better capture features of PDAC micro- and macro- environments to inform patient stratification for tailored treatments.

**Box 1. Glossary**

**2D cells:** GEMM- or patient-derived PDAC cell lines are cultured in 2D/monolayer *in vitro*.

**2D cell-derived mouse models:** PDAC 2D cells that have been cultured in monolayer are injected into mice.

**Genetically engineered mouse models (GEMMs):** Mice have their genome edited to spontaneously develop PDAC.

**Intrasplenic metastatic mouse models:** Typically used to model liver metastases. PDAC 2D cells or organoids are injected into the spleen. Optionally, the spleen, or part of the spleen, can then be removed.

**Isogenic organoids:** PDAC organoids that have been engineered *in vitro* (e.g. using CRISPR/Cas9) to only differ for a single genetic event (e.g. *Smad4* loss).

**Organoids:** GEMM- or patient- derived PDAC organoid lines are cultured in a 3D matrix (e.g. Matrigel) *in vitro*.

**Organoid-derived mouse models:** PDAC organoids (isogenic or not) that have been cultured in a 3D matrix are injected into mice.

**Orthotopically grafted mouse models:** PDAC 2D cells or organoids are injected into the pancreas.

**Xenograft mouse models:** Patient-derived PDAC 2D cells or organoids are injected into mice.

**Subcutaneous mouse models:** PDAC 2D cells or organoids are injected into the mouse subcutaneous layer, typically in the flank.

**Syngeneic mouse models:** Murine PDAC 2D cells or organoids (e.g. GEMM-derived) are injected into mice.

**Tail-vein metastatic mouse models:** Typically used to model lung metastases. PDAC 2D cells or organoids are injected into the tail vein.

## Modelling the micro-environment: genetics, stroma and metastases

### Heterogenous genetics

The malignant cell compartment of human PDAC is heterogeneous. Molecular subtyping based on tumour transcriptional profiles has been proposed as a way to stratify patients (Collisson et al., 2011; Moffitt et al., 2015; Bailey et al., 2016). However, this classification has not translated into different therapeutic regimens, possibly owing to co-existence of these subtypes within the same tumour (Chan-Seng-Yue et al., 2020; Raghavan et al., 2021; Williams et al., 2023). An alternative strategy could be based on mutational profiles, and targeting of specific genetic drivers is increasingly available (Golan et al., 2019; Hosein et al., 2022; Kemp et al., 2023; Strickler et al., 2023). Four genes are predominantly altered in patient tumours and are the main drivers of PDAC progression: *KRAS*, *TP53*, *CDKN2A* and *SMAD4* (Raphael et al., 2017; Aguirre et al., 2018).

We propose that the lack of pre-clinical models that recapitulate diverse genetics as well as the assumption that findings in KRAS^G12D^/mutant p53 tumours from KPC mice are generalisable to other contexts contribute to the high failure rate of translating pre-clinical observations to the clinic.

*Kras^LSL-G12D/+^*; *Trp53^LSL-R172H/+^*; *Pdx1-Cre* − also known as Kras, p53,Cre (KPC) mice − are the most broadly used genetically engineered mouse models (GEMMs) of PDAC and recapitulate the development of the human disease (Hingorani et al., 2005). However, only a small percentage of patient tumours are solely characterised by KRAS^G12D^ and mutant p53 (Raphael et al., 2017; Aguirre et al., 2018). Other GEMMs, such as KC (*Kras^LSL-G12D/+^; Pdx1-Cre*) mice, facilitate research of the early stages of the disease (Hingorani et al., 2003). Finally, less commonly studied GEMMs have been developed to model additional patient-relevant mutations, including *Cdkn2a* or *Smad4* loss (Bardeesy et al., 2006; Izeradjene et al., 2007; Whittle et al., 2015). However, more-complex GEMMs are lacking as they would be time-consuming and expensive to maintain, which impedes their development and widespread utilisation. We propose that the lack of pre-clinical models that recapitulate diverse genetics as well as the assumption that findings in KRAS^G12D^/mutant p53 tumours from KPC mice are generalisable to other contexts contribute to the high failure rate of translating pre-clinical observations to the clinic.

PDAC organoids (Box 1) derived from GEMMs or patients recapitulate the genetics of the primary tumour (Boj et al., 2015; Tiriac et al., 2018), undergo less genomic evolution than 2D cells (Ben-David et al., 2019) and can be easily genetically modified to reflect a range of mutations as isogenic organoids (Box 1). Moreover, compared to GEMMs, mice orthotopically grafted with PDAC organoids (Box 1) facilitate more-rapid modelling of distinct mutational profiles (Biffi et al., 2019; Miyabayashi et al., 2020). Finally, comparison of isogenic organoid-derived mouse models (Box 1) can effectively pinpoint therapeutic vulnerabilities that depend on a specific genetic event.

One disadvantage of using PDAC organoids derived from GEMMs is that the commonly used strains provide a limited representation of patient tumour genetics. Moreover, in some cases, modelling the progressive accumulation of mutations could be important to elucidate mechanisms of PDAC progression, and normal pancreatic organoids could be sequentially mutated using CRISPR, base editing and/or prime editing strategies (Komor et al., 2016; Gaudelli et al., 2017; Anzalone et al., 2019) prior to transplantation. Alternatively, these strategies could be directly applied in mice (Gürlevik et al., 2016; Sánchez-Rivera et al., 2022). Finally, new GEMMs that enable sequential activation of different driver mutations over time will also be important, although their widespread application across laboratories might not be feasible.

Expanding the repertoire of models with different genetic profiles could be instrumental to informing effective therapies that target malignant features of PDAC as well as the complex tumour-promoting stroma.

### Extensive stroma

Human PDAC has an extensive and heterogenous non-malignant stroma that shapes PDAC progression and therapy response. Thus, PDAC patient stratification based on malignant cell-stroma interdependencies may be a more effective strategy than current classifications. Indeed, evidence suggests that distinct mutations in PDAC malignant cells can differentially influence the tumour microenvironment (TME) (Laklai et al., 2016; Vennin et al., 2019; Maddalena et al., 2021; Shaashua et al., 2022). Specifically, cancer-associated fibroblasts (CAFs) are abundant and heterogeneous cell types within the PDAC stroma (Öhlund et al., 2017; Elyada et al., 2019; Biffi and Tuveson, 2020; Dominguez et al., 2020; McAndrews et al., 2022). Malignant cell-dependent reprogramming and distinct cells of origin have been shown to contribute to CAF heterogeneity and function (Öhlund et al., 2017; Biffi et al., 2019; Biffi and Tuveson, 2020; Garcia et al., 2020; Helms et al., 2021; Hutton et al., 2021). However, additional models for lineage-tracing, as well as for genetic manipulation of specific PDAC CAF subtypes are needed. Indeed, understanding CAF heterogeneity is essential for dissecting their different roles in PDAC progression to design effective stromal-targeting therapeutic agents.

GEMMs and mice orthotopically grafted with PDAC organoids can faithfully recapitulate PDAC stroma heterogeneity (Boj et al., 2015; Biffi et al., 2019; Miyabayashi et al., 2020). It is worth noting that patient-derived material needs to be transplanted into immunocompromised mice (Pham et al., 2021). Thus, due to the lack of a complete immune system, only a partial evaluation of therapy response can be made in these xenograft mouse models (Box 1), although current humanized mouse models can be implemented to overcome some of these limitations (Hye Jeong et al., 2023). However, as several murine ligands do not bind to human receptors, adequate analysis and targeting of bi-directional malignant cell-stroma crosstalk in xenograft mouse models (Box 1) will require co-transplantation of additional patient-derived stromal cell types. Finally, we urge PDAC researchers to stop using 2D cell-derived (either orthotopic or subcutaneous) and subcutaneous (either 2D cell- or organoid- derived) mouse models (Box 1) – even if they are syngeneic mouse models (Box 1) – for pre-clinical therapeutic studies, as they inadequately recapitulate CAF composition and immune cell infiltration (Erstad et al., 2018; Biffi and Tuveson, 2020).

Faithfully modelling the TME is required for the design of effective therapies. As most PDAC patients present with advanced disease, this consideration is important not only in primary tumours but also at metastatic sites.

### Predominant metastatic disease

PDAC patients with metastases are not eligible for surgery. For these patients, successful treatments need to target both the pancreatic tumour and the metastases. However, pre-clinical evaluation of drug efficacy is often only based on primary tumour growth. Moreover, our understanding of the differences between primary tumours and metastases − in terms of genetics, stroma composition and therapy − remains limited (Aiello et al., 2016). Indeed, on the one hand, most PDAC GEMMs, such as KPC mice, will succumb because of primary tumour-associated complications rather than metastases, hindering in-depth analyses of these sites. On the other hand, tail-vein or intrasplenic metastatic mouse models (Box 1) prevent a comparison to matched primary tumours (O'Brien et al., 2023; Lücke et al., 2024).

New pre-clinical models of PDAC that better recapitulate the patient experience and late-stage disease are needed. This includes development of more highly metastatic GEMMs that, for example, model genetic events known to increase metastasis (Rahrmann et al., 2022), implementation of surgical strategies for resection of the primary tumour (Gürlevik et al., 2016) and evaluation of drug efficacy on both primary tumours and metastases.

## Modelling the macro-environment: comorbidities and risk factors

### Ageing and cancer-associated cachexia

The incidence of PDAC increases with age, with the highest rates occurring in men and women at the ages of 65-69 years and 75-79 years, respectively (Pourshams et al., 2019). Despite this, commonly used pre-clinical mouse models − including KPC mice − are typically ∼2-6 months old, which corresponds to ∼20-30 human years (Wang et al., 2020). Thus, these models might fail to recapitulate the physiological state and micro-environmental features of most PDAC cases. For example, the aged TME shapes cancer progression in other malignancies (Fane and Weeraratna, 2020; Marino and Weeraratna, 2020). Moreover, PDAC patients of different age groups present distinct molecular features (Ben-Aharon et al., 2019; Tsang et al., 2021; Ogobuiro et al., 2023). However, age-dependent micro-environmental changes in PDAC, and their effect on PDAC progression and therapy response, are yet to be fully understood.

Additionally, older adults can develop sarcopenia, a condition defined by depletion of muscle mass and impaired physical performance (Cruz-Jentoft et al., 2019). However, so far, the interplay between sarcopenia and systemic effects promoted by PDAC − including cancer-associated cachexia, a comorbidity with symptoms closely related to sarcopenia (e.g. muscle loss) − is largely unknown (Dunne et al., 2019; Kordes et al., 2021). Importantly, cancer-associated cachexia is partially driven by TME factors (Ferrer et al., 2023), and analysis of old PDAC model mice suggests differences in these factors compared to young mice (Dasgupta et al., 2022).

The development of patient-relevant aged mouse models of PDAC will reveal new crosstalk between ageing, PDAC progression, comorbidities and therapy response. Findings in these models may lead to patient stratification based on micro-environmental and macro-environmental age-specific changes.

### Obesity, type 2 diabetes and chronic pancreatitis

In addition to ageing, other PDAC-associated risk factors, including obesity, type 2 diabetes (T2D) and chronic pancreatitis, promote disease development and alter therapy response (Klein, 2021). However, the molecular mechanisms linking these factors to progression of PDAC remain largely unknown. Recently, diet- or genetically induced obese mouse models of PDAC have started to better characterise the impact of obesity on PDAC progression (Chung et al., 2020; Kfoury et al., 2022). In addition, obesity can drive a metabolic syndrome that contributes to the onset of T2D (Shen et al., 2023). Recent studies have investigated the contribution of T2D to PDAC initiation and progression by using diabetic mouse models and show that T2D generates a more-permissive environment for malignant cells to grow (Wang et al., 2016; Velazquez-Torres et al., 2020). Moreover, mouse models of chronic pancreatitis have highlighted the role of prolonged inflammation in driving PDAC progression (Reed and Gorelick, 2014; Merry and Petrov, 2018). However, efforts should be made to improve current chronic pancreatitis-inducing strategies to mouse models that are more patient-relevant. For example, recapitulating the irreversible changes associated with chronic pancreatitis may require a combination of GEMMs that spontaneously develop this disease and pancreatitis-inducing agents, such as the cholecystokinin analogue caerulein (Lerch and Gorelick, 2013; Merry and Petrov, 2018).

Since macro-environmental risk factors can impact PDAC initiation, progression and therapy response, pre-clinical models that recapitulate them could reveal their impact on micro-environmental features and guide the design of new combination therapies. These models could also be leveraged to discover early-detection biomarkers and to guide screening strategies for high-risk groups.

## Conclusion

Considering PDAC as multiple diseases and, in accordance, modelling the heterogeneity of its micro- and macro- environments will guide patient stratification, screening strategies and the development of effective therapeutics.

**Fig. 1.**
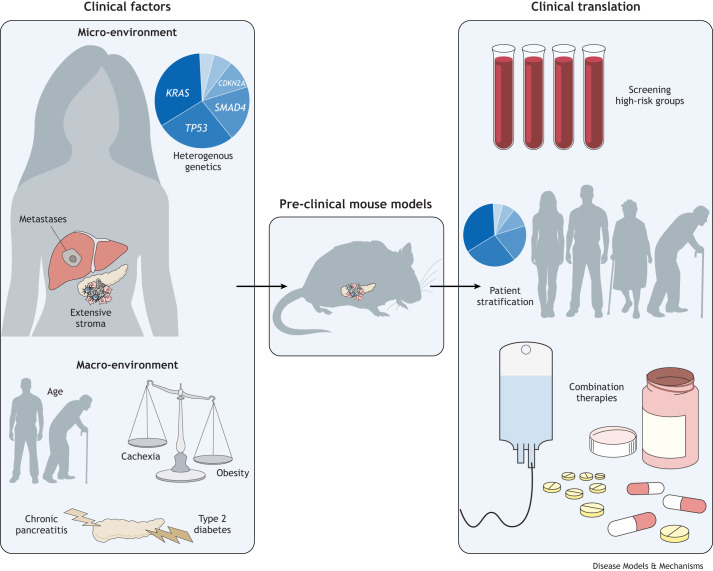
**Modelling the complexity of PDAC micro- and macro- environments towards more effective clinical translation.** PDAC is characterised by complex clinical factors. Key PDAC micro-environment features include diverse malignant cell mutational profiles, an extensive non-malignant stroma, and a predominantly metastatic disease. In addition to these micro-environment features, the PDAC macro-environment consists of a multitude of associated risk factors and comorbidities that influence cancer progression and therapy response, including cancer-associated cachexia, ageing, obesity, type 2 diabetes and chronic pancreatitis. Capturing PDAC complexity in pre-clinical mouse models will inform screening of high-risk groups, patient stratification and effective combination therapies.

We believe that a shift in mind-set and strategy is required for developing the next generation of PDAC treatments and diagnostics. Successful translational research will need to move away from oversimplifying the inherent complexity of PDAC. Considering PDAC as multiple diseases and, in accordance, modelling the heterogeneity of its micro- and macro- environments will guide patient stratification, screening strategies and the development of effective therapeutics (Fig. 1). Importantly, PDAC complexity cannot be deconvoluted and tackled in isolation. Instead, it will require a collaborative and coordinated effort of researchers sharing cross-disciplinary expertise as well as complex and − consequentially − more-expensive, pre-clinical models. This will require funding bodies to not only support ambitious multi-laboratory projects but to also help create the infrastructure needed for effective collaborations. Altogether, these synergistic efforts will help drive meaningful pre-clinical discoveries for successful clinical translation and improved PDAC survival.

**Figure:**
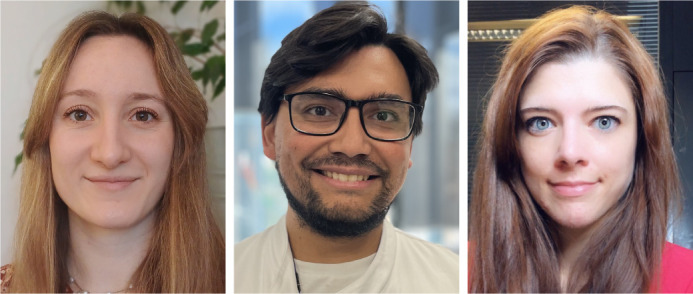
Eloise G. Lloyd, Joaquín Araos Henríquez (middle) and Giulia Biffi (left to right)
